# Hope and Loneliness as Predictors of Quality of Life Among Rural Older Adults in Thailand: A Cross-Sectional Study

**DOI:** 10.3390/ijerph22081189

**Published:** 2025-07-29

**Authors:** Bovornpot Choompunuch, Naphat Wuttaphan, Wipanee Suk-erb

**Affiliations:** 1 Department of Educational Psychology and Guidance, Faculty of Education, Mahasarakham University, Maha Sarakham 44000, Thailand; bovornpot.c@msu.ac.th; 2 Research Unit of Interdisciplinary and Lifelong Learning, Faculty of Education, Mahasarakham University, Maha Sarakham 44000, Thailand; 3 Department of Human Resource and Organization Management, Faculty of Management Science, Pibulsongkram Rajabhat University, Phitsanulok 65000, Thailand; naphat.w@psru.ac.th

**Keywords:** hope, loneliness, quality of life, older adults, rural Thailand, psychosocial health

## Abstract

Hope and loneliness are significant psychosocial factors that greatly influence the quality of life (QoL) among older adults. However, few studies have examined these constructs simultaneously in rural aging populations in Southeast Asia. This study aimed to investigate the relationships between hope, loneliness, and QoL among community-dwelling older adults in northeastern Thailand. A cross-sectional study was conducted with 250 participants aged 60 years and older, recruited through convenience sampling. Descriptive statistics summarized participant characteristics, while hierarchical regression identified QoL predictors. The participants (mean age = 70.41 years; 52.8% female) reported a high level of hope (M = 33.35), a moderate level of loneliness (M = 8.81), and a good level of QoL (M = 99.13). Hierarchical regression analysis revealed that age, occupation, monthly income, income source, education, health insurance, comorbidities, hope, and loneliness were significant predictors of QoL. Together, these factors accounted for 55.1% of the variance in QoL. Both hope and loneliness have a significant impact on QoL in older adults. Interventions designed to reduce loneliness and foster hope may prove effective in enhancing the well-being of aging populations. These findings underscore the importance of integrating psychosocial and community-based approaches into geriatric care and public health planning.

## 1. Introduction

As the global population ages, ensuring the well-being of older adults has become an increasingly vital focus for public health. In Thailand, people aged 60 and older constitute more than 19.5% of the overall population, with the most significant demographic shifts occurring in rural areas [1]. Although aging can provide valuable opportunities for personal development, it also brings forth various challenges that can affect physical, psychological, and social well-being [2]. Specifically, older adults frequently face heightened risks of social isolation, loneliness, and a lower quality of life (QoL), particularly in community settings with limited resources [3].

The QoL for older adults is influenced by various factors, including their health, financial situation, social networks, and psychological traits such as hope and feelings of loneliness [4]. Hope, which is a forward-looking, goal-oriented mindset, has been demonstrated to enhance psychological well-being and improve coping skills in later life [5]. In contrast, loneliness—characterized by a perceived lack of meaningful social interaction—has been repeatedly associated with an increased likelihood of depression, cognitive deterioration, and higher mortality rates among older adults [6].

Resilience theory, founded in positive psychology, views psychological strengths, such as hope, as dynamic resources that enable individuals to maintain or recover their well-being in the face of stress [7]. Research conducted on older adults in rural areas has indicated that resilience is bolstered by mental health and social connections [8,9]. However, there is a lack of empirical research on how specific constructs like hope operate in rural Thai contexts. Likewise, studies from other low- and middle-income countries suggest that while broader indicators of self-esteem and social support are associated with QoL [10], the combined impact of hope and loneliness—particularly within cultural contexts like that of Thailand [11]—has not been thoroughly investigated.

Thailand’s cultural landscape is marked by strong family ties and a robust community-oriented approach, which serve as protective factors against loneliness and foster a sense of hope among its citizens [12]. In rural areas, however, the scenario is complex due to limited access to healthcare services, a shortage of mental health professionals, and a heavy reliance on family and community networks for care [9]. Older adults in these regions frequently encounter transportation challenges and a heightened risk of social isolation, making it more difficult to obtain necessary psychosocial support compared to their urban counterparts [2]. Recent studies suggest an increasing prevalence of older adults living alone in rural settings, which exacerbates feelings of social isolation [13]. While national research has highlighted various factors such as physical health and financial stability as key determinants of QoL, there remains a notable gap in the literature concerning the inclusion of positive psychological aspects like hope and resilience [14,15]. Addressing these factors may ultimately provide a more holistic understanding of QoL for older adults in rural Thailand.

The existing literature on QoL among older adults in Thailand presents a notable gap, particularly regarding the psychological constructs of hope and loneliness in rural communities. While various studies have documented the physical, socioeconomic, and psychological factors affecting QoL, the specific influence of hope and loneliness remains underexplored. These constructs were chosen for this study due to their significant empirical links to emotional well-being in older populations. Hope and loneliness, unlike constructs such as resilience or self-efficacy, are especially susceptible to social changes and support systems inherent in aging rural cohorts. The scarcity of research addressing the interplay between these factors is particularly striking, given that resilience theory posits an interaction between them that can impact adaptive aging. Therefore, this study aimed to investigate the association between hope, loneliness, and QoL among community-dwelling older adults in a rural province of northeastern Thailand. The research has three specific objectives: (1) to evaluate the levels of hope, loneliness, and QoL among community-dwelling older adults, (2) to examine the relationships between hope, loneliness, and QoL, and (3) to identify the socio-demographic and psychosocial factors that predict QoL in this population. By identifying key psychosocial predictors of QoL, the findings aim to contribute to the broader discourse on aging well and to inform policy and practice in geriatric care and community health promotion. Findings from this study may inform community-based interventions, such as village elder clubs, religious counseling services, and mobile mental health outreach programs, to promote psychosocial well-being in rural older adults.

## 2. Theoretical Framework

This study is grounded in resilience theory, a conceptual framework from positive psychology that emphasizes individuals’ capacity to maintain or regain psychological well-being in the face of adversity [7]. Resilience is understood as a dynamic process involving the interaction between personal strengths and external environmental supports, allowing individuals to adapt positively despite stress, trauma, or chronic challenges [16]. In the context of aging, resilience is a critical determinant of health and QoL. Older adults often face complex stressors, including declining physical health, reduced income, bereavement, and social isolation [2]. According to resilience theory, two significant psychosocial variables—hope and loneliness—serve as crucial mediators in this adaptation process [16].

Hope is conceptualized as a cognitive-motivational construct comprising agency (goal-directed energy) and pathways (planning to achieve goals) [5]. It promotes positive adaptation by enabling older adults to envision meaningful futures, maintain psychological strength, and pursue personal goals despite adversity. Within resilience theory, hope acts as a protective factor, enhancing emotional regulation, social engagement, and proactive coping strategies [17,18]. Empirical evidence supports the notion that hope improves health behaviors, mitigates depressive symptoms, and increases life satisfaction among older adults [19,20].

Conversely, loneliness is recognized as a psychosocial risk factor that undermines resilience, disrupts emotional security, and diminishes social capital [21]. It is defined as the subjective perception of insufficient social connection and is associated with an increased risk of mental and physical illness, cognitive decline, and even mortality [21,22]. Within the resilience framework, persistent loneliness weakens adaptive capacity by undermining perceived support networks and reducing one’s sense of belonging and purpose in life.

The relationship between hope and loneliness is particularly significant among aging populations, where changes in social roles, declining health, and reduced mobility can either foster or hinder resilience. Resilience theory suggests that hope can mitigate the harmful effects of loneliness, acting as a psychological mechanism that helps older adults reinterpret or cope with social disconnection [23,24].

In rural Thai communities, these dynamics are further shaped by cultural factors such as collectivist values, filial piety, and Buddhist beliefs. While these cultural traditions often create a protective environment by promoting intergenerational support and spiritual coping, modernization, and youth migration are leading to increased isolation and role changes among older individuals [11,25]. Consequently, rural older adults may experience heightened psychosocial stress, making resilience—specifically the interaction of hope and loneliness—a critical lens for examining their overall QoL.

**Figure 1:**
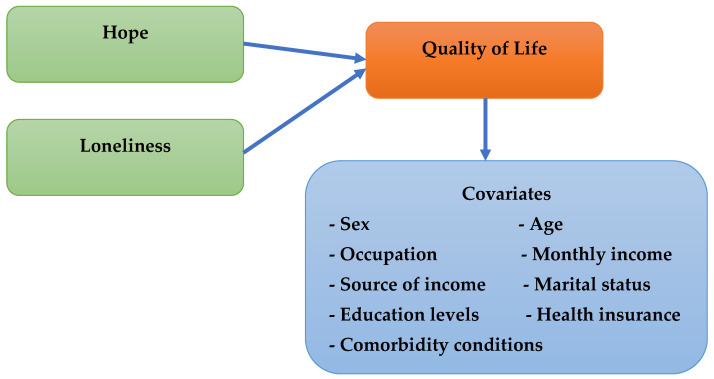
Conceptual framework.

Thus, this study integrates resilience theory to investigate how hope (as a protective factor) and loneliness (as a risk factor) influence QoL, considering contextual variables like aging, health status, and social structure. Guided by this framework, we hypothesize that (H1) higher levels of hope are positively associated with better QoL among rural older adults; (H2) higher levels of loneliness are negatively associated with QoL; and (H3) socio-demographic factors such as monthly income and health status significantly influence QoL in this population (see Figure 1).

## 3. Materials and Methods

### 3.1. Research Design and Sampling

**Figure 2:**
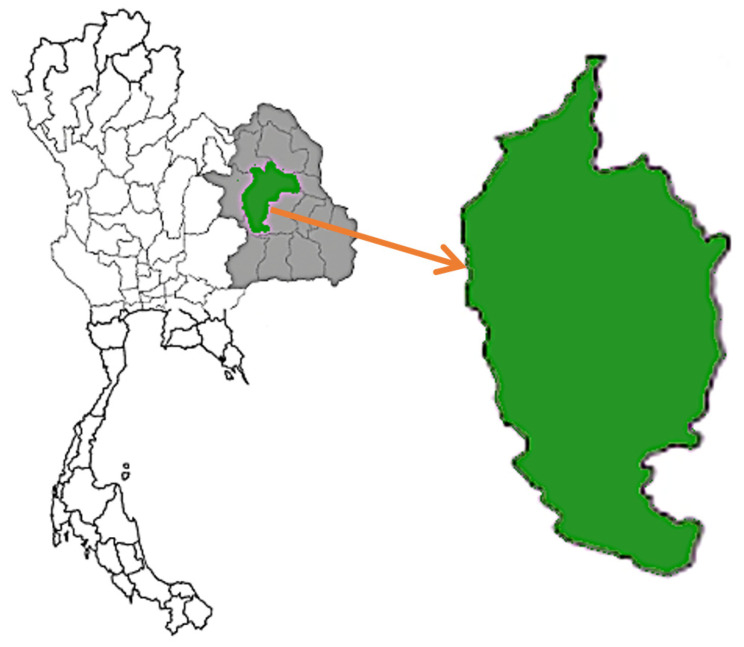
A map of the study site highlighting the Maha Sarakham Province (green), the area where primary data collection was conducted.

This cross-sectional study was conducted in Mahasarakham Province, northeastern Thailand, where individuals aged 60 and above account for 20.13% of the population [26]. A map of the study area and comparative demographic data (see Figure 2) underscore the region’s relevance as a representative rural setting. Nationally, the aging population in Thailand accounts for 19.5%, which is slightly above the global average [1]. This site was selected for its representativeness of rural aging populations and strong participation in local community health initiatives. Participants were selected through convenience sampling; although practical for accessing older adults in community settings, the use of convenience sampling may limit generalizability due to potential selection bias and underrepresentation of individuals who are socially isolated or less active in community life. The eligibility criteria for participation included: (1) individuals aged 60 years and older, regardless of gender; (2) those residing within the community; (3) participants without a history of cognitive impairment, Alzheimer’s disease, or mental health disorders; and (4) individuals capable of understanding and communicating in Thai. Exclusions applied to individuals with other visual, auditory, or sensory impairments, those diagnosed with a mental health disorder, and anyone unwilling to participate voluntarily. Participants with sensory impairments or diagnosed mental disorders were excluded to minimize confounding effects and to ensure that all participants could independently complete the self-administered questionnaires. The sample size was calculated using the G*Power program (version 3.1) [27,28]. An effect size of 0.15 was taken from a prior study [29], with a significance level set at 0.05 and a power of 0.8. To accommodate potential incomplete responses in the questionnaires, the sample size was increased by approximately 30%. As a result, the total sample size for this study was 250 participants.

In this research, QoL served as the primary dependent variable and was evaluated using the WHOQOL-OLD instrument, which assesses various dimensions of well-being specific to older adults. The principal independent variables were hope and loneliness. Hope was conceptualized as a protective psychological factor aligned with resilience theory, while loneliness—assessed via the UCLA Loneliness Scale—was treated as a psychosocial risk factor negatively associated with QoL. To account for potential confounding influences, a range of sociodemographic variables were included directly in the hierarchical regression analysis. These variables included age, sex, marital status, education level, monthly income, source of income, health insurance, and comorbidity conditions.

### 3.2. Research Instruments

Hope Scale (HS): The HS measures hope among community-dwelling older adults. The scale developed by Wongsinudom [30] consists of 10 items and utilizes a 4-point response format (1 = strongly disagree, 4 = strongly agree). Total scores can range from 10 to 40, categorized as low level (<11), moderate level (11–30), and high level (>30), with higher scores indicating greater levels of hope. In this study, the Cronbach’s alpha coefficient was measured at 0.83.

UCLA Loneliness Scale (RULS-6): The RULS-6 was employed to assess loneliness among participants. This 6-item instrument has been translated and tested by Wongpakaran, et al. [31]. We utilized the Thai version of the RULJS-6 in this study. Responses are rated on a scale from 1 (never) to 4 (always) across three categories: emotional loneliness (three questions) and social loneliness (three questions). A higher score indicates greater feelings of loneliness, with total scores ranging from 6 to 24. These scores are categorized as follows: <7 (low), 7–18 (moderate), and >18 (high). The study reported a Cronbach’s alpha coefficient of 0.83, indicating good internal consistency.

World Health Organization Quality of Life-OLD (WHOQOL-OLD): The WHOQOL-OLD was used to evaluate older adults’ QoL. This instrument was developed by Power, et al. [32]. In 2007, it was translated into Thai through a specific transcultural adaptation method Sudnongbua [33]. The assessment comprises 24 items divided into six domains, with four items pertaining to each of the following dimensions: sensory abilities, autonomy, past, present, and future, social participation, death and dying, and intimacy. Each item has five response options on a five-point Likert scale. Total scores range from 24 to 120 and are categorized as follows: poor QoL (<57), moderate QoL (57–90), and good QoL (>90). The Cronbach’s alpha coefficient in this study was 0.84.

Sociodemographic Questionnaire: We included sociodemographic information with nine questions: sex, age, occupation, monthly income, source of income, marital status, education levels, health insurance, and comorbidity conditions.

### 3.3. Data Collection

After receiving approval from the Human Research Ethics Committee, all participants were given a comprehensive explanation of the study’s goals, methods, and measures in place to safeguard their rights. Before the data collection started, written informed consent was obtained. Participants were instructed on how to fill out the questionnaires and were encouraged to ask questions or voice any concerns at any time. Questionnaires were administered in person, individually, at participants’ homes or local community centers by the researcher team. The format was face-to-face, and assistance was provided as needed for participants with low literacy skills. Once the questionnaires were completed, each set of responses was meticulously checked for completeness and accuracy. Any missing or unclear responses were promptly addressed by the participants to maintain data quality. Only fully validated datasets were used for statistical analysis.

### 3.4. Data Analysis

Descriptive statistics—including frequencies, percentages, means, and standard deviations—were employed to summarize the sociodemographic characteristics of the participants. Following this, hierarchical regression analysis was conducted to identify key predictors of QoL among community-dwelling older adults. Predictor variables were introduced in theoretically informed blocks (e.g., sociodemographic controls followed by psychological variables), facilitating an assessment of the incremental variance explained at each stage. Prior to regression modeling, all standard assumptions were evaluated and confirmed to be satisfied: normality of residuals, linearity, absence of multicollinearity (with Variance Inflation Factors below 5), and no significant autocorrelation. Graphical diagnostics and VIF metrics were consulted as needed to verify these conditions. Statistical significance was established at an alpha level of *p* < 0.05 for all analyses, and effect sizes (ΔR^2^ for model blocks) were interpreted to assess practical significance. All analyses were performed using SPSS Version 26.

## 4. Results

This study involved 250 participants, most of whom were female (52.80%), with an average age of 70.41 years (SD = 7.78). Among them, 138 participants (55.20%) were in the 60 to 69 age range. The most common occupation was agriculture, accounting for 33.60% of the sample. Additionally, 68% of participants indicated a monthly income of less than 10,000 Thai Baht, and 36.40% depended on the elderly living allowance. A significant majority identified as married (63.60%) and had educational attainment at the primary school level or below (82.40%). In terms of healthcare coverage, 90.80% were enrolled in the universal health coverage scheme, while 58.80% reported having one or more comorbid conditions (See Table 1).

### 4.1. Loneliness, Hope, and Quality of Life

Participants exhibited moderate loneliness, with a mean score of 8.78 (SD = 3.04), split into 4.44 (SD = 1.80) for emotional loneliness and 4.37 (SD = 1.72) for social loneliness. Hope levels were high, with a mean score of 33.35 (SD = 4.37), comprising agency (16.16, SD = 2.77) and pathway (17.19, SD = 2.32) scores. QoL was rated good at 99.13 (SD = 11.90), with autonomy, social participation, death and dying, and intimacy also rated positively. However, sensory abilities and past, present, and future activities were moderate, scoring 15.75 (SD = 3.11) and 14.80 (SD = 2.37), respectively (see Table 2).

### 4.2. Factors Influencing QoL Among Older Adults

In this study, the factors influencing the prediction of QoL were examined through hierarchical regression analysis. As demonstrated in Table 3, several significant predictors of QoL were identified among community-dwelling older adults: age (β = −0.110, *p* = 0.012), occupation (β = 0.152, *p* = 0.004), monthly income (β = −0.130, *p* = 0.017), source of income (β = 0.185, *p* < 0.001), education levels (β = 0.198, *p* = 0.001), health insurance (β = −0.172, *p* = 0.001), comorbidity conditions (β = −0.197, *p* < 0.001), hope (β = 10.631, *p* < 0.001), and loneliness (β = −5.325, *p* < 0.001). Collectively, these variables accounted for approximately 55.1% of the variance in QoL.

## 5. Discussion

This research explored the relationship between hope, a positive psychological asset, and loneliness, an indicator of social disconnection, in relation to QoL among older adults in rural Thailand. The results indicated that higher levels of hope were linked to improved QoL, while increased loneliness was associated with QoL. This pattern aligns with resilience theory, which suggests that personal strengths can mitigate the negative impact of stress on well-being [34].

Loneliness, often referred to as a “geriatric giant,” can harm mental health and lead to feelings of hopelessness in later life [35]. In contrast, hope and similar positive beliefs serve as protective factors, aiding older individuals in coping with life’s challenges and enhancing their overall life satisfaction [17,36]. Overall, our findings support the idea that resilience arises from the interplay between psychological resources and social disconnection: even in situations of limited social interaction, maintaining a hopeful perspective can uphold QoL [15], whereas persistent loneliness can weaken one’s sense of hope and purpose [24]. These insights provide a deeper understanding of aging through the lens of resilience, highlighting the importance of interventions that address both individual strengths and social contexts to promote healthy aging.

The present study found that higher levels of hope were significantly associated with better QoL among rural older adults. This finding aligns with prior studies suggesting that hope serves as a protective psychological factor promoting emotional well-being and resilience. Hope-based interventions have shown promise in improving well-being among older adults [18,37]. However, few studies have evaluated such interventions in Southeast Asian or Thai contexts. Adapting these approaches to reflect local beliefs, collectivist values, and Buddhist worldviews could enhance their relevance and effectiveness. For example, a prior study has developed a monk-led mental health counseling program grounded in Buddhist traditions, demonstrating improved skills and acceptability among Thai older adults [38]. This suggests that culturally sensitive interventions aimed at fostering could be a promising strategy to enhance the quality of life in this population.

Our findings align with trends observed in both global and regional studies on aging populations. A substantial amount of research indicates that loneliness has a markedly adverse effect on the well-being and QoL of older adults [39]. For instance, longitudinal studies conducted in Europe have shown that initial levels of loneliness can predict decreases in QoL in the following years [35]. Likewise, cross-sectional studies conducted in various contexts consistently reveal that older people who are socially isolated or lonely experience poorer health, higher levels of depression, and lower life satisfaction [40,41].

In Southeast Asia, where populations are experiencing significant demographic aging, worries about loneliness are increasing. According to the World Health Organization [42], the number of individuals aged 60 and over in this region is expected to rise from 77 million in 2020 to 173 million by 2050, underscoring the need to prioritize the social and emotional well-being of older populations. Recent statistics suggest that loneliness impacts a considerable percentage of older adults in Asia; for example, approximately 32.5% of older adults in Malaysia [43] and 31.7% in Myanmar have reported feelings of loneliness [44]. Moreover, local surveys in specific rural areas of Thailand found that around one-quarter of elderly individuals experience loneliness [13]. Our findings suggest that loneliness is negatively associated with hope, although causality cannot be inferred due to the cross-sectional design.

Simultaneously, the protective function of hope that we identified aligns with growing evidence from positive psychology related to older adults. Research involving older adults in the United States [20] and Asia [19] has established a connection between higher levels of hope and increased life satisfaction, optimism, and even healthier lifestyle choices. A significant longitudinal study conducted in the United States indicated that a heightened sense of hope during late life is associated with improved psychosocial outcomes (such as less sleep disturbance, increased physical activity, and enhanced life satisfaction) [17]. Similarly, findings from a study on older adults in Singapore revealed that a supportive family environment can enhance hope while mitigating feelings of loneliness, thus leading to greater happiness and life satisfaction [25].

Interestingly, our findings revealed a statistically significant negative association between monthly income and QoL (β = −0.130, *p* = 0.017). While this contrasts with previous research indicating a positive link between income and QoL, several possible explanations may account for this divergence. In rural Thai communities, higher income may be associated with increased family expectations, caregiving burdens, or financial stressors—such as debt or remittance responsibilities—which have been linked to poorer quality of life among caregivers in Northeastern Thailand (adjusted odds ratio for poor QoL with debt = 5.89; 95% CI 3.85–9.01) [45]. Alternatively, subjective well-being among older adults may be more influenced by non-material resources, such as family support, health, or community engagement. For example, greater social capital—particularly perceived support and neighborhood safety—has been associated with higher health-related QoL among rural older Thai adults (OR = 1.87, 95% CI 1.10–3.17) [22].

The findings of this study support key tenets of resilience theory, particularly the role of hope as a psychological asset that enhances individuals’ capacity to cope with life stressors and maintain well-being [16]. Our results demonstrated that greater levels of hope were significantly associated with higher QoL among rural Thai older adults, consistent with studies in both Western and Asian populations [2,46]. Hope may serve as a mediating mechanism that encourages proactive coping strategies, emotional regulation, and goal persistence, even in the face of limited financial or healthcare resources [47]. Conversely, loneliness—a known psychosocial risk factor—was inversely related to QoL, suggesting that social disconnection impairs resilience by eroding social capital and diminishing perceived meaning in life [21]. Additionally, a prior systematic review emphasizes that resilience frameworks can inform interventions designed to enhance coping mechanisms within older populations through structured social and cognitive activities [48]. Interventions informed by this framework—such as group-based positive-thinking programs that incorporate reflection sessions, discussions, and homework assignments—have shown significant improvements in both resilience and life satisfaction among community-dwelling older adults [36].

In rural Thai contexts, these strategies could be effectively implemented by village health volunteers (VHVs), who play a pivotal role in community-based mental health promotion [49]. Utilizing culturally adapted formats—such as guided reflection that incorporates Buddhist metaphors, peer support circles led by VHVs, or monastic-assisted well-being workshops—would resonate with local values and religious traditions [38]. Empirical evidence supports that group-based, volunteer-led interventions enhance social cohesion and resilience among rural populations, especially during times of crisis [50]. Therefore, the integration of structured group activities, guided reflection, and culturally sensitive hope-enhancement therapies delivered by VHVs presents a promising model for improving psychological resilience and quality of life among older adults in rural areas [51].

The cross-cultural similarities highlighted suggest that hope and social connections are essential factors impacting the QoL of older adults, regardless of contextual differences. Our research provides unique insights from a rural region in Southeast Asia to the global literature, reinforcing the widely acknowledged connection between loneliness and a diminished QoL. Furthermore, it highlights the importance of hope as a vital and universally significant component of resilience throughout the aging process.

Among the demographic variables analyzed, only sex and marital status were not significantly associated with QoL in this study. While many studies have found associations between marital status or gender and QoL in older adults, the lack of significance here may reflect sociocultural characteristics specific to rural Thai communities. For example, older adults in these areas often maintain strong ties with extended family regardless of marital status, and receive consistent support from children, relatives, or community networks. As such, being widowed or single may not carry the same degree of social or emotional disadvantage as observed in more urbanized or individualistic contexts [52,53]. Similarly, gender roles among rural Thai elders may converge in later life due to shared caregiving responsibilities, participation in religious or communal activities, and similar access to social resources, which could diminish gender-based disparities in QoL [54].

### 5.1. Strengths and Limitations of the Study

A significant advantage of this study is its emphasis on older adults living in rural areas of a developing country, a demographic that is frequently overlooked in gerontological research. By investigating psychosocial well-being in rural Thailand, we address a geographic gap and provide relevant evidence regarding aging populations in Southeast Asia. The research also benefited from the use of established measurement tools and incorporates insights from recent literature to frame the connection between hope, loneliness, and quality of life. However, it is important to recognize several limitations. Firstly, the cross-sectional nature of the study restricts our ability to infer causation. While we found associations that align with theoretical expectations and previous longitudinal studies, we cannot definitively assert that loneliness led to a decrease in QoL or that hope mitigated this impact. There may be bidirectional influences at play (for instance, low QoL could also intensify feelings of loneliness), and conducting longitudinal follow-ups would enhance our ability to interpret causal relationships. Secondly, since all data on hope, loneliness, and QoL were self-reported, there is a risk of response biases. Cultural aspects in Thailand—such as a hesitance to acknowledge feelings of loneliness or a preference for positive self-presentation—may have contributed to underreporting of psychosocial distress. Thirdly, the sample was sourced from a single rural area, which could influence the extent to which findings can be generalized. Older individuals in rural Thailand often experience different life circumstances (for example, more traditional lifestyles and the out-migration of younger family members) compared to their urban counterparts or those in other nations [13]. Caution is advised when extrapolating our conclusions to dissimilar community settings. Despite these limitations, the research offers valuable insights by linking psychological resilience (hope) and social health (loneliness) to QoL among a rural elder population in a non-Western context. Future studies could build upon this groundwork through longitudinal designs and wider sampling—such as comparing rural and urban demographic groups—to further explore the underlying mechanisms. Fourthly, this study has a relatively small sample size, which may limit the generalizability of the findings. As the sample was drawn from a single rural province using a convenience sampling method, the results may not reflect the experiences of older adults in other regions or urban settings. This limitation highlights the need for caution in interpreting the findings and underscores the importance of conducting future research with larger and more representative samples. This study is limited by its reliance on quantitative data, which may not fully capture the subjective meaning of ‘hope’ and ‘loneliness’ as experienced by older adults in rural Thailand. Future research should consider a mixed-methods design, incorporating qualitative interviews or focus groups to explore cultural interpretations and emotional nuances that might provide deeper insights into how older adults perceive hope and deal with loneliness in their everyday lives. By acknowledging both the strengths and limitations of our study, we accurately position our findings and highlight avenues for future exploration. Additionally, while structural equation modeling techniques such as PLS-SEM may have been suitable for exploring complex mediating pathways, the current sample size and cross-sectional design limited our ability to use such advanced analysis. Future studies with larger samples are encouraged to apply these methods.

### 5.2. Policy Implications and Community-Based Recommendations

This research emphasizes the necessity of tackling both social isolation and psychological strength to enhance QoL for older adults, especially in rural and resource-limited environments. Effective strategies involve expanding social engagement initiatives—such as befriending programs, senior clubs, and intergenerational activities—to combat feelings of loneliness and foster connection. Mental health services should incorporate hope-boosting methods like goal-setting therapy and meaning-centered interventions, which have been proven to enhance emotional resilience and well-being in later life. Thailand’s initiative of “Elderly Schools” serves as a culturally relevant example of this approach, providing older adults with opportunities to learn, engage, and rediscover a sense of purpose. Expanding such models in partnership with local organizations (e.g., temples, community centers) could significantly decrease loneliness and improve psychosocial health in rural regions. Additionally, improved hope and reduced loneliness have been linked in other studies to increased health service utilization, better chronic disease management, and reduced depressive symptoms—indicating their relevance to overall health and functioning in aging populations [20].

Additionally, healthcare systems should implement integrated care models that cater to both medical and psychosocial needs. Regular screenings for feelings of loneliness and hopelessness by primary care providers and community health workers can assist in the early identification of individuals at risk. These initiatives should be complemented by mobile units or age-friendly centers that provide medical checkups along with social and psychological services. Successful execution will necessitate collaboration across multiple sectors, including government agencies, NGOs, and local leaders. Investing in mental health and social infrastructure for the elderly can yield significant long-term benefits—not only by lowering healthcare expenses but also by enhancing dignity, connection, and purpose for aging populations. Our findings emphasize the importance of cultivating both social connectedness and internal hope to promote healthy and fulfilling aging.

## 6. Conclusions

This study contributes to the growing body of research emphasizing the role of psychosocial factors—particularly hope and loneliness—in shaping the QoL among older adults in rural, under-resourced settings. Our findings suggest that loneliness is a significant negative predictor of QoL, while hope serves as a crucial protective factor that may buffer the emotional and social challenges associated with aging. This relationship highlights the potential of fostering hope as a form of psychological resilience, particularly in contexts where social connectedness is limited. These results carry important implications for public health and geriatric care in Thailand and similar cultural settings. Culturally tailored, community-based initiatives aimed at reducing loneliness and enhancing hope could support the psychological well-being and life satisfaction of aging populations. Interventions delivered by trained community health workers or village health volunteers may be especially effective in reaching older adults in rural areas. However, the findings must be interpreted with caution due to the study’s relatively small and regionally specific sample. As such, the results may not be generalizable to broader or more diverse populations. Future research should employ larger, representative samples and consider longitudinal or mixed methods designs to further explore the causal pathways between hope, loneliness, and QoL. Additionally, evaluating the effectiveness of hope-focused and social support interventions across various aging communities could offer deeper insights into strategies for promoting resilience and well-being among older adults.

## Figures and Tables

**Table 1:** Sociodemographic Status of the Participants.

Demographic Characteristics		Frequency (n)	Percent (%)
Sex			
	Female	132	52.80
	Male	118	47.20
Age (Mean ± SD)		70.41 ± 7.78	
	60–69	138	55.20
	70–79	71	28.40
	>80	41	16.40
Occupation			
	Agriculture	84	33.60
	General employee	44	17.60
	Business	52	20.80
	Government employee	13	5.20
	Unemployed	57	22.80
Monthly Income (Thai baht)			
	<10,000	170	68.00
	10,000–15,000	61	24.40
	>15,000	19	7.60
Source of Income			
	Family member	57	22.80
	Part-time job	89	35.60
	Pension	13	5.20
	Elderly living allowance	91	36.40
Marital status			
	Sigle	32	12.80
	Married	159	63.60
	Window	29	23.60
Education Levels			
	Primary school or lower	206	82.40
	High school	32	27.80
	Undergrads or higher	12	4.80
Health Insurance			
	Universal health coverage	227	90.80
	Social security	13	5.20
	Civil servant	10	4.00
Comorbidity conditions			
	Yes	147	58.80
	No	103	41.20

**Table 2:** The Mean, Standard Deviation of Loneliness, Hope, and Quality of Life Scores.

Variables		Mean	SD	Interpretation
Loneliness		8.81	3.04	Moderate
	Emotional loneliness	4.44	1.80	Moderate
	Social loneliness	4.37	1.72	Moderate
Hope		33.35	4.37	High
	Agency or Willpower	16.16	2.77	High
	Pathway or Way power	17.19	2.32	High
Quality of life		99.13	11.90	Good
	Sensory abilities	15.75	3.11	Moderate
	Autonomy	16.86	3.19	Good
	Past, present, and future activities	14.80	2.37	Moderate
	Social participation	17.08	2.37	Good
	Death and dying	17.40	2.55	Good
	Intimacy	17.24	2.38	Good

**Table 3:** Hierarchical regression of risk factors for QoL among older adults.

Model	B	SE (b)	β	t	*p*-Value
Model 1					
(Constant)	4.565	0.220		20.763	<0.001
Sex	0.054	0.061	0.054	0.875	0.383
Age	−0.111	0.040	−0.168	−2.815	0.005
Occupation	0.024	0.018	0.091	1.299	0.195
Monthly Income	−0.087	0.059	−0.110	−1.476	0.141
Source of Income	0.063	0.027	0.151	2.311	0.022
Marital status	−0.042	0.054	−0.050	−0.771	0.441
Education Levels	0.236	0.074	0.246	3.203	0.002
Health Insurance	−0.130	0.077	−0.115	−1.681	0.094
Comorbidity conditions	−0.312	0.066	−0.308	−4.740	<0.001
R = 0.416, R^2^ = 0.173, Adjusted R^2^ = 0.142, R^2^ change 0.173, F = 5.586, *p*-value < 0.001					
Model 2					
(Constant)	1.927	0.262		7.368	<0.001
Sex	0.048	0.047	0.048	1.022	0.308
Age	−0.076	0.030	−0.115	−2.507	0.013
Occupation	0.054	0.014	0.206	3.788	<0.001
Monthly Income	−0.085	0.045	−0.106	−1.873	0.062
Source of Income	0.077	0.021	0.185	3.718	<0.001
Marital status	−0.007	0.041	−0.008	−0.171	0.864
Education Levels	0.136	0.057	0.142	2.400	0.017
Health Insurance	−0.168	0.059	−0.149	−2.850	0.005
Comorbidity conditions	−0.223	0.051	−0.221	−4.406	<0.001
Hope	0.724	0.055	0.636	13.146	<0.001
R = 0.721, R^2^ = 0.520, Adjusted R^2^ = 0.500, R^2^ change 0.347, F = 25.908, *p*-value < 0.001					
Model 3					
(Constant)	2.629	0.281		9.367	<0.001
Sex	0.032	0.044	0.033	0.730	0.466
Age	−0.073	0.029	−0.110	−2.545	0.012
Occupation	0.040	0.014	0.152	2.902	0.004
Monthly Income	−0.103	0.043	−0.130	−2.411	0.017
Source of Income	0.077	0.020	0.185	3.910	<0.001
Marital status	0.032	0.040	0.038	0.806	0.421
Education Levels	0.189	0.055	0.198	3.463	0.001
Health Insurance	−0.195	0.056	−0.172	−3.462	0.001
Comorbidity conditions	−0.199	0.048	−0.197	−4.138	<0.001
Hope	0.604	0.057	0.530	10.631	<0.001
Loneliness	−0.252	0.047	−0.257	−5.325	<0.001
R = 0.756, R^2^ = 0.751, Adjusted R^2^ = 0.551, R^2^ change 0.051, F = 28.826, *p*-value < 0.001					

## Data Availability

The datasets generated and analyzed in the current study are not publicly available online because the university has copyright. The datasets used during the current study are available from the corresponding author upon reasonable request.

## Abbreviation

The following abbreviations are used in this manuscript:

QoL	Quality of Life
